# The Role of Canonical Transient Receptor Potential Channels in Seizure and Excitotoxicity

**DOI:** 10.3390/cells3020288

**Published:** 2014-04-09

**Authors:** Fang Zheng, Kevin D. Phelan

**Affiliations:** 1 Department of Pharmacology and Toxicology, University of Arkansas for Medical Sciences, 4301 West Markham Street, Slot 611, Little Rock, AR 72205, USA; 2 Department of Neurobiology and Developmental Sciences, University of Arkansas for Medical Sciences, 4301 West Markham Street, Slot 846, Little Rock, AR 72205, USA; E-Mail: phelankevind@uams.edu

**Keywords:** canonical transient receptor potential channels, metabotropic glutamate receptors, epileptiform burst firing, seizure, excitotoxicity

## Abstract

Canonical transient receptor potential (TRPC) channels are a family of polymodal cation channels with some degree of Ca^2+^ permeability. Although initially thought to be channels mediating store-operated Ca^2+^ influx, TRPC channels can be activated by stimulation of Gq-coupled G-protein coupled receptors, or by an increase in intracellular free Ca^2+^ concentration. Thus, activation of TRPC channels could be a common downstream event of many signaling pathways that contribute to seizure and excitotoxicity, such as N-methyl-D-aspartate (NMDA) receptor-mediated Ca^2+^ influx, or metabotropic glutamate receptor activation. Recent studies with genetic ablation of various TRPC family members have demonstrated that TRPC channels, in particular heteromeric TRPC1/4 channels and homomeric TRPC5 channels, play a critical role in both pilocarpine-induced acute seizures and neuronal cell death. However, exact underlying mechanisms remain to be fully elucidated, and selective TRPC modulators and antibodies with better specificity are urgently needed for future research.

## 1. Introduction

Seizures arise from the synchronized burst firing of a large group of cortical neurons. Seizures can be induced by repetitive electric stimulations (*i.e*., kindling) of the perforant pathway in the hippocampus, or by chemical convulsants (e.g., the muscarinic agonist pilocarpine) that act on the hippocampal circuitry resulting in synchronized cortical firing. The hippocampus contains some of the neuronal populations that are most vulnerable to excitotoxicity, which is neuronal cell death caused by over-activation of glutamate receptors [1,2]. 

Excitotoxicity has emerged as a shared pathophysiological component of stroke, epilepsy and head trauma [2,3]. Minutes after the onset of ischemia, ATP in the severely affected core region is depleted, and all ATP-dependent processes that are critical to maintain normal function stop. Similarly, the depletion of ATP also occurs during intense seizures. Loss of ATP-dependent membrane transport results in the collapse of ion gradients across cell membranes thereby initiating a vicious cycle: accumulation of extracellular K^+^ leads to membrane depolarization, which in turn exacerbates energy deficits and further destabilizes ion gradients. The collapse of ion gradients also impairs clearance of glutamate from the synaptic cleft which floods the extracellular space with glutamate leading to widespread neuronal cell death.

Excessive Ca^2+^ influx is thought to trigger excitotoxicity [2]. The N-methyl-D-aspartate (NMDA) receptor, a subtype of ionotropic glutamate receptor with high calcium permeability, is thought to play a key role and was the first to be targeted for stroke therapy [4]. Although supported by many studies in animal models [4], clinical trials with NMDA antagonists all failed [5,6] suggesting that mechanisms underlying clinically relevant excitotoxicity are not yet fully understood. There are likely alternative signaling pathways leading to Ca^2+^ overload such as those involving metabotropic glutamate receptors (mGluRs), a family of G-protein coupled glutamate receptors [7]. 

## 2. The Role of Group I mGluRs in Seizure and Excitotoxicity

The group I mGluRs coupled to phospholipase C (*i.e*., mGluR1 and mGluR5) have been implicated in seizure and excitotoxicity [8,9,10]. The first mGluR selective agonist, (1S,3R)-1-aminocyclopentane-1,3-dicarboxylic acid (1S,3R-ACPD), induced limbic seizures and neuronal degeneration *in vivo* [8]. A selective group I mGluR agonist, (S)-3,5-dihydroxyphenylglycine (DHPG), had the same effect [10] indicating that the seizures and neuronal cell death induced by the broad-spectrum mGluR agonist 1S,3R-ACPD are likely mediated by group I mGluRs). The pattern of neurodegeneration induced by 1S,3R-ACPD [9] was similar to the neurodegeneration induced by kainic acid [11]. Two of the most vulnerable regions to the cell death caused by 1S,3R-ACPD are the hippocampal CA3 region and the lateral septum, both of which are highly vulnerable to limbic seizures induced by electrical kindling [12]. 

Why are the hippocampal CA3 region and lateral septum highly vulnerable to limbic seizures and excitotoxicity? In lateral septal neurons, mGluR agonists induce “epileptiform” burst firing with a large depolarizing plateau potential [13,14] that is similar to the “paroxysmal depolarization shift”. The plateau potential was triggered by membrane depolarization and was not blocked by tetrodotoxin [15]. Group I mGluR agonists induce similar burst firing in CA3 pyramidal neurons [8,16,17]. Under voltage-clamp recording, activation of mGluRs results in an inward current permeable to both sodium and calcium, with a negative slope region in their I-V relationship [18,19,20]. Therefore, this plateau potential is thought to be mediated by a Ca^2+^-activated non-selective (CAN) current. Inward membrane currents with a negative slope become greater in amplitude following membrane depolarization and subsequently generate additional depolarization. This positive feedback loop is capable of forming a self-regenerative plateau potential underlying the epileptiform burst firing observed in lateral septal neurons. Furthermore, the CAN-current in lateral septal neurons is a major source of Ca^2+^ influx that likely contributes to excitotoxicity [21].

The molecular identity of ion channels mediating the CAN current remained uncertain for years but the canonical transient receptor potential (TRPC) channels have emerged recently as the leading candidate [22]. The aim of this review is to summarize the recent data that support a role of TRPC channels in seizure and excitotoxicity and discuss the critical issues that need to be resolved in future studies. 

## 3. Expression, Structure and Pharmacology of TRPC Channels

TRPCs are the mammalian homologues of drosophila *trp* channels. There are seven members (TRPC1-7) in the mammalian TRPC family (for review, see [23]). All but one of the TRPC genes have been identified in the human genome (TRPC2 is a pseudogene) and may contribute to various human diseases. Based on sequence homology and functional properties, the TRPC family can be divided into two subgroups: TRPC1/4/5 and TRPC3/6/7.

Structurally, the superfamily of TRP channels share a common architecture with voltage-gated potassium channels and calcium channels. All of the TRP family members have six putative transmembrane regions and a putative reentrance loop that forms the ion-conducting pore [24]. The transmembrane regions are flanked by an intracellular amino-terminal domain and an intracellular carboxyl-terminal domain. Ion channels with this type of architecture are typically tetrameric, *i.e*., formed by four subunits. A recent study of TRPV1 supports the structural similarity between the *trp* channels and potassium channels [25] but the structure of TRPC channels remains to be determined. 

At the present time, our knowledge regarding the expression of TRPC channels in the central nervous system has relied largely on *in situ* hybridization of TRPC mRNAs. Among 7 members of the TRPC family, TRPC1 is ubiquitously expressed while other TRPC family members have a more discrete expression pattern in the brain [26,27]. Moderate to high expression of TRPC1 can be detected in most limbic areas. Expression of TRPC2, a pseudogene in humans, is mainly limited to the olfactory bulbs in rodents [26,27]. TRPC3 expression is most prominent in the cerebellum [26,28,29]. TRPC4 is highly expressed in the lateral septum and the CA1 region of the hippocampus [30,31,32,33]. TRPC5 is highly expressed in the CA1-CA3 region of the hippocampus and the amygdala [34,35]. TRPC6 is highly expressed in the dentate gyrus [36]. TRPC7 (originally named TRP7) mRNA has been detected at moderate to low levels in the olfactory bulbs, cerebellum, and dentate gyrus [37]. Commercial antibodies against various members of the TRPC family have not all been validated using TRPCKO mice. The specificity of commercial antibodies against TRPC4 has been questioned [38]. A commercial TRPC7 antibody (Santa Cruz) is actually generated specifically against a TRP now known as TRPM2 [39]. The lack of specific antibodies at the present time is a huge roadblock for the study of neuronal TRPC channels, and there is an urgent need for the development and specificity validation of antibodies against most TRPC family members.

All TRP channels are cation channels with various permeability ratios for calcium and sodium. Functional TRPC channels are thought to be either homomeric or heteromeric channels formed by members in the same subgroup [35,40,41]. For example, TRPC1 can form homomeric channels by itself or heteromeric channels with TRPC4 or TRPC5 in an artificial expression system. However, interactions between TRPCs from different subgroups have been reported [42,43]. These reported interactions need to be viewed with skepticism until the specificity of the TRPC antibodies are confirmed using TRPC knockout mice. The exact gating mechanism for TRPC channels remains hotly debated. TRPC1, 4 and 5 channels have been proposed to function as store-operated channels [26,30,34,44], and they interact directly with stim1, a recently identified molecular sensor for store depletion [45]. However, recent studies have shown that the gating of TRPC channels comprised of TRPC1/4/5 is “polymodal” since they can be activated by stimulation of G-protein coupled receptors, such as mGluR1 [46], or by a rise of intracellular free Ca^2+^ directly [22,47]. Importantly, this calcium sensitivity allows TRPC channels comprised of TRPC1/4/5 to amplify calcium signaling. TRPC3, 6, and 7 channels, on the other hand, do not directly interact with stim1 [45] but are instead directly activated by diacylglycerol [48]. In summary, there are many controversies and questions regarding the subunit composition and functional properties of TRPC channels. Data obtained from artificial expression systems need to be assessed in native biological systems using genetic knockout approaches.

Few selective drugs for TRPC channels have been discovered. SKF96365 inhibits all TRPC channels, but is considered to be nonspecific since it also inhibits other ion channels [49,50]. La^3+^ blocks most TRPC channels with the exception of channels containing TRPC4 or TRPC5. Acting on three glutamate residues shared by TRPC4 and TRPC5, La^3+^ activates the homomeric TRPC4 and TRPC5 channels and heteromeric TRPC1/5 channels [35,51]. Presumably, heteromeric TRPC1/4 channels should also be potentiated by La^3+^. However, the native TRPC4 current that was eliminated in TRPC4 knockout (KO) mice was blocked by low micromolar La^3+^ [52]. A recent study has suggested that ML204 is a selective inhibitor of TRPC4 [53], however, it is ineffective against native TRPC4 containing channels in lateral septal neurons [54]. Pyr-3 has been reported as a selective inhibitor of TRPC3 channels [55] but its selectivity remains to be confirmed in TRPC3KO mice. Recent drug screening studies have identified a few compounds that apparently act against TRPC6 [56,57,58,59,60] though only one of these compounds (hyperforin) has been tested in TRPC6 KO mice with apparent mixed results. Ding et al [59] demonstrated the absence of hyperforin induced constriction of aortic segments in TRPC6KO mice, while Chen *et al*. [60] reported attenuation, but not complete blockade of hyperforin induced microvascular leakage in TRPCKO mice. In summary, there are no drugs that target TRPC channels with confirmed specificity that would allow them to be used as pharmacological tools to investigate the functional roles of specific TRPC channels. At the present time, functional studies of neuronal TRPC channels must rely on targeted genetic ablation. Global disruption of a gene through development could have unintended consequences. The use of inducible or tissue-specific genetic approaches would be preferable.

## 4. TRPC Channels, the Ca^2+^-Activated Non-Selective (CAN) Current and Epileptiform Burst Firing in the Lateral Septum

**Figure 1:**
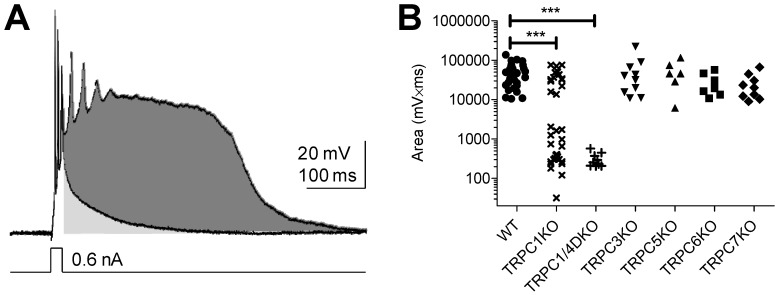
Quantitative comparison of the 1S,3R-ACPD-induced plateau potential in lateral septal neurons in WT and TRPC KO mice. (**A**) Representative traces showing the normal decay of the membrane potential after spikes generated by a brief depolarizing current pulse under control conditions and the plateau potential after spikes generated by the same depolarizing current pulse following bath perfusion of 30 µM 1S,3R-ACPD. The area-under-the curve (area; shaded) was measured from the end of the depolarizing current pulse to a time point after the end of the plateau potential (typically 1 or 2 s). The mean of total area (shaded with both light and dark gray) in the presence of 1S,3R-ACPD for each lateral septal neuron was plotted in B; (**B**) The total area in the presence of 1S,3R-ACPD in WT, TRPC1KO, TRPC1/4DKO, TRPC3KO, TRPC5KO, TRPC6KO, TRPC7KO (*n =* 34, 36, 9, 10, 6, 7, 9 respectively). Note that the TRPC1KO and TRPC1/4DKO groups were significantly different from the WT group (***: *p <* 0.001, Kruskal-Wallis test and Dunn's Multiple Comparison Test). Traces in A adapted from Figure 3B of [33]. The WT, TRPC1KO and TRPC1/4DKO data in B are replotted from the raw data presented in Figure 3C of [33]. The TRPC3KO, TRPC5KO and TRPC6KO data were reported in [33]. TRPC7KO data is unpublished data that was obtained in a similar manner.

As mentioned earlier, the expression level of TRPC4 is the highest in the lateral septum, a limbic relay nucleus. In lateral septal neurons, mGluR agonists induce “epileptiform” burst firing with a large depolarizing plateau potential similar to the “paroxysmal depolarization shift” [13,14]. To determine whether this plateau potential is mediated by TRPC channels, we and others recorded from lateral septal neurons in a panel of TRPC KO mice [33,54]. The plateau potential is altered in TRPC1KO mice and is totally absent in TRPC4KO and TRPC1/4 double knockout (DKO) mice, but is normal in TRPC3KO, TRPC5KO, TRPC6KO, and TRPC7KO mice (Figure 1) [33]. These observations indicate the involvement of TRPC1 and TRPC4 in the mGluR1/5-mediated plateau potential and rule out the involvement of TRPC3, 5, 6, and 7. Our data also suggest that the TRPC channels responsible for the plateau potential are likely heteromeric TRPC1/4 channels. 

It should be noted that the mGluR agonist-induced plateau potential is present in a small group of lateral septal neurons in TRPC1KO mice, suggesting that homomeric TRPC4 channels are capable of mediating the plateau potential [33]. The remaining question is why a plateau potential is only observed in a small group of lateral septal neurons. It appears that the presence of TRPC1 may be critical to the coupling between mGluR1/5 and TRPC channels though the exact molecular mechanism for such a requirement remains to be determined. 

Although sharing sensitivity to SKF96365, the pharmacological properties of TRPC channels that mediate the plateau potential in lateral septal neurons appear to be distinct from the homomeric recombinant TRPC4 channels. First, La^3+^ exhibited only inhibitory effects on the plateau potential in lateral septal neurons [61] whereas it potentiates recombinant homomeric TRPC4 channels. Second, ML204, a reported selective inhibitor of recombinant homomeric TRPC4 channels, yielded inconsistent results against the plateau potential in lateral septal neurons ([54] which was attributed to possible degradation of ML204 in aqueous solutions. However, our personal experience suggests that this is not the case. We observed that in a single lateral septal neuron, ML204 blocks the plateau potential at 20 µM but is ineffective at 50 µM, suggesting complex pharmacological actions of ML204 on heteromeric TRPC1/4 channels [61]. Further investigation of the pharmacological properties of native TRPC1/4 channels in lateral septal neurons is needed to reach a definitive conclusion.

**Figure 2:**
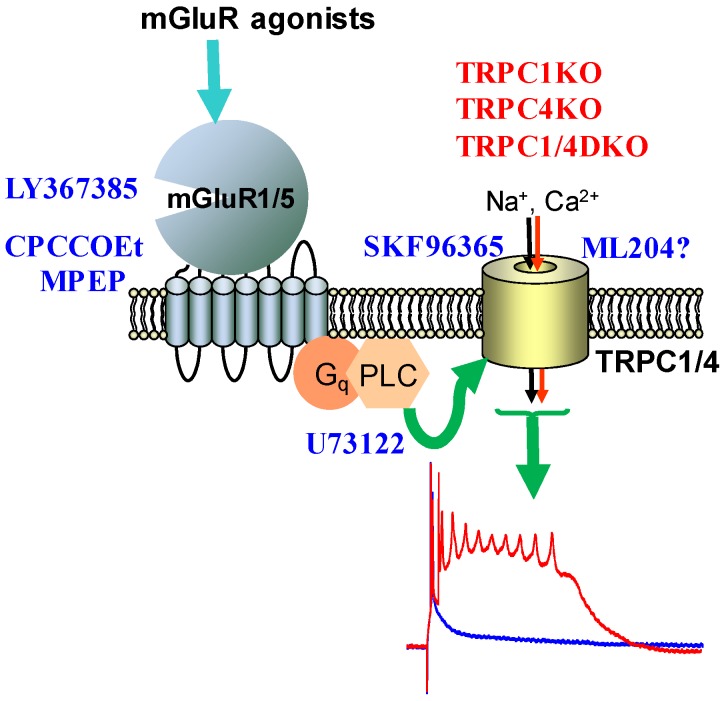
The signaling cascade leading to epileptiform burst firing in lateral septal neuron.

Figure 2 summarizes our current understanding regarding the underlying molecular mechanisms of mGluR agonist-induced epileptiform burst firing in lateral septal neurons. Group I mGluRs (mGluR1 and mGluR5) are both involved since specific negative allosteric modulators of mGluR1 and mGluR5 (CPCCOEt and MPEP, respectively) [62] can abolish the epileptiform burst firing induced by DHPG or 1S,3R-ACPD. It is possible that mGluR1 and mGluR5 may form heterodimers in lateral septal neurons since CPCCOEt and MPEP can block mGluR agonist-induced bursting in the same lateral septal neuron [33]. Downstream of activation of mGluR1 and 5, activation of phospholipase C (PLC) is also required because the epileptiform burst can be abolished by U73122, a selective PLC inhibitor [63]. Heteromeric TRPC1/4 channels are then activated by this signaling cascade, leading to the plateau potential underlying the epileptiform burst.

## 5. TRPC Channels and Epileptiform Burst Firing in the Hippocampus

Kandel and Spencer [64] observed three types of firing patterns in hippocampal pyramidal neurons: single spikes, short bursts, and long-lasting bursts (100–200 ms). The long-lasting bursts were regarded as the cellular equivalents of interictal discharges and are defined as epileptiform burst firing. There has been a long standing debate about the underlying mechanism for the plateau potential associated with epileptiform burst firing [65]: some investigators suggest that it is generated by a neural network and requires activation of NMDA receptors; others suggest that it reflects a change in intrinsic membrane properties and is mediated by a persistent Na^+^ current [66], low-threshold voltage-gated calcium channels [67,68], or a CAN current [69]. Since TRPC4 and 5 are well expressed in the hippocampus, TRPC channels comprised of TRPC1/4/5 may play a critical role in epileptiform burst firing.

We recorded CA1 pyramidal neurons using the current-clamp intracellular recording method, and induced spontaneous epileptiform burst firing by bath application of 1S,3R-ACPD, a mGluR agonist known to induce seizures *in vivo*. We showed that in TRPC1KO mice and TRPC1/4DKO mice, mGluR agonist-induced spontaneous burst firing in CA1 pyramidal neurons was significantly reduced [70], and what remained were short bursts with an average of 3 spikes, which may be mediated by T-type or R-type voltage-gated Ca^2+^ channels [67,68]. In contrast, TRPC5KO mice exhibit normal mGluR agonist-induced epileptiform burst firing [70]. This came as a surprise since several groups suggested that TRPC5 contributes to the plateau potential [71,72,73]. Thus, our data suggest that TRPC1/4 channels play a critical role in the epileptiform burst firing in CA1 pyramidal neurons paralleling the functional role of these channels in lateral septal neurons as summarized in Figure 2.

Using the same panel of TRPC knockout mice, we also examined the role of TRPC1/4 channels in the epileptiform burst firing in CA3 pyramidal neurons. Spontaneous epileptiform burst firing can be induced in CA3 pyramidal neurons by bath application of bicuculline for 30 min [74] or by high-frequency stimulation of the mossy fiber pathway [75]. Although Wong and colleagues have proposed that the CAN current is the underlying mechanism for the plateau potential in CA3 pyramidal neurons [17], others have shown that the burst firing is synaptic in origin and reflects enhanced synaptic strength at recurrent collateral synapses via NMDA receptor-dependent long-term potentiation (LTP) [74,75]. TRPC5 may play a critical role in the mGluR5-dependent enhancement of NMDA-receptor-dependent LTP at CA3 recurrent collateral synapses. Future studies are needed to validate this hypothesis and pinpoint the exact role of TRPC5 in the generation of epileptiform burst firing in CA3 pyramidal neurons.

## 6. The Role of TRPC1, 4, 5 in Seizure and Excitotoxicity *in Vivo*

**Figure 3:**
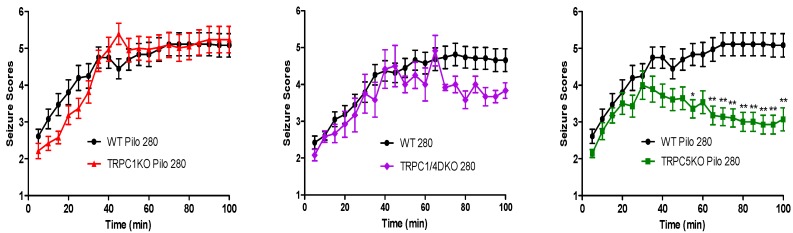
Pilocarpine-induced seizures were significantly reduced in TRPC5KO mice. The time course of pilocarpine-induced seizures in WT, TRPC1KO, TRPC1/4DKO, and TRPC5KO mice after a single injection of pilocarpine (280 mg/kg, i.p.). Pooled data (mean ± SEM) was plotted (*n =* 18, 14, 6, 19 for WT, TRPC1KO, TRPC1/4DKO and TRPC5KO mice). See Phelan *et al*. 2012 for description of seizure scale. Note significantly reduced seizure scores in TRPC5KO mice at the late phase after pilocarpine injection (***: *p <* 0.001, Two-way ANOVA; *: *p <* 0.05; **: *p <* 0.01, Bonferroni *post hoc* tests against WT). The TRPC1KO and TRPC5KO data are adapted from Figure 2 of [70], while the TRPC1/4KO data is from Figure 4A of [33].

The full extent and range of potential contribution of TRPC channels to seizure and excitotoxicity can only be assessed *in vivo*. We studied the role of TRPC channels in seizure susceptibility and seizure-induced neuronal cell death, using the pilocarpine-induced *status epilepticus* model (Figure 3). The dramatic reduction of epileptiform burst firing at the cellular level in CA1 in TRPC1KO and TRPC1/4DKO mice failed to lead to a reduction of pilocarpine-induced acute seizures [33]. On the other hand, the normal epileptiform bursting in CA1 in TRPC5KO *in vitro* is associated with a significant reduction in the severity of pilocarpine-induced seizures *in vivo* [70]. Since TRPC1KO mice and TRPC1/4DKO mice show normal severity of pilocarpine-induced seizures (Figure 3), homomeric TRPC5 channels are likely involved in pilocarpine-induced acute seizures, not heteromeric channels such as TRPC1/4, TRPC1/5 or TRPC1/4/5. 

**Figure 4:**
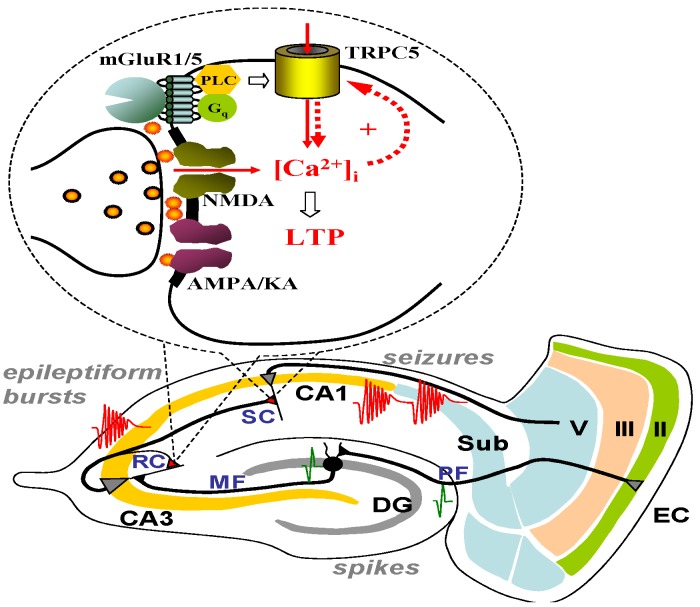
A working hypothesis for TRPC5 in seizure and excitotoxicity. We propose that TRPC5 is critical for mGluR1/mGluR5-mediated enhancement of NMDA receptor-dependent long-term potentiation at SC synapses in CA1 and RC synapses in CA3. The enhanced LTP at SC synapses contributes to hyperexcitability and excitotoxicity in CA1 while enhanced LTP at RC synapses in CA3 contributes to the initiation of seizure and excitotoxicity in CA3. EC: entorhinal cortex; DG: dentate gyrus; MF: mossy fiber; RC: recurrent collaterals; SC: Schaffer collaterals; Sub: subiculum.

The underlying mechanism of TRPC5 contribution to pilocarpine-induced acute seizures has yet to be elucidated. Our current hypothesis is that homomeric TRPC5 channels located in or near dendritic spines, are activated by Group I mGluRs and amplify the dendritic calcium signals thereby enhancing NMDA receptor-dependent LTP (Figure 4). This mechanism would apply to both Schaffer collateral-CA1 synapses and CA3 recurrent collateral synapses. Alternatively, TRPC5 channels may directly contribute to the plateau potential underlying spontaneous epileptiform burst firing in CA3 pyramidal neurons. TRPC5 channels may also be expressed at the presynaptic terminals and linked to neurotrophic factor receptors [76] where they play a role in synaptic plasticity. More intriguingly, there is evidence that the surface expression of TRPC5 may be dynamically regulated [72]. It is possible that the pilocarpine-induced acute seizures may result from increased surface expression of TRPC5 channels within different components of the cortical circuitry. Future studies are needed to test these hypotheses and elucidate the underlying mechanisms of TRPC5’s role in acute seizures.

**Figure 5:**
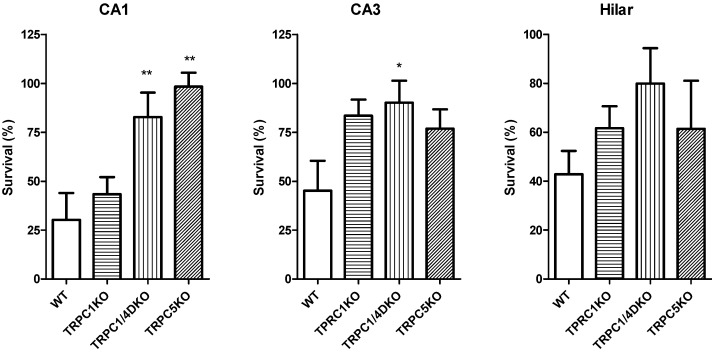
Neuronal cell death after pilocarpine-induced seizures in the hippocampus of WT, TRPC1KO, TRPC1/4DKO and TRPC5KO mice. Neuronal cell death induced by pilocarpine-induced seizures was assessed in selected groups of mice with comparable average seizure scores above three for WT (3.79 ± 0.14, *n =* 6), TRPC1KO (3.89 ± 0.13, *n =* 6), TRPC1/4DKO (3.89 ± 0.20, *n =* 5), and TRPC5KO mice (3.45 ± 0.10, *n =* 5). Serial coronal sections (50 µm) from mice with similar pilocarpine-induced seizures were stained with Nissl and surviving neurons (with stained cytoplasm and round nuclei) were counted using Stereologer with a 100X oil-immersion objective. The percentage of surviving neurons was calculated by dividing the cell count in each pilocarpine-treated mouse with the averaged cell count in WT control mice (mice without seizures). There was no significant difference in the number of neurons between WT and untreated KO mice. Pooled data (mean ± SEM) were plotted. Note the significant increase in surviving neurons in the CA1 region of TRPC1/4DKO and TRPC5KO (**: *p <* 0.01, ANOVA), and the significant increase in surviving neurons in the CA3 region of TRPC1/4DKO (*: *p <* 0.05, ANOVA). Adapted from data presented in Figure 6 of [33] and Figure 4 of [70] relative to untreated WT mice.

Figure 5 summarizes the extent of pilocarpine-induced neurodegeneration in different subfields of the hippocampus. A previous study suggested that TRPC1 plays a critical role in glutamate-induced excitotoxicity in the hippocampus in slice cultures [77]. However, we failed to detect any significant reduction in pilocarpine-induced neuronal cell death in the hippocampus in TRPC1KO mice. The seizure-induced neuronal cell death is reduced significantly in the CA1 and lateral septum in TRPC1/4DKO mice [33], and this reduction corresponds well with the epileptiform burst firing mediated by TRPC1/4 channels in those brain regions [33]. Pilocarpine-induced neuronal cell death in TRPC5KO mice is also significantly reduced in the CA1 area, but there is some variability in the CA3 area. It should be noted that the neuronal cell death in the hilar region is not significantly reduced by genetic ablation of TRPC1, 4 or 5. 

## 7. Do TRPC3, 6, or 7 Play a Role in Seizure and Excitotoxicity?

This review has mostly focused on the role of the TRPC1/4/5 subgroup in seizure-induced neuronal cell death. The contribution of TRPC3/6/7 subgroup to seizure and excitotoxicity has yet to be fully elucidated, but there is some evidence in favor of such a role. Neuronal TRPC3 channels, for example, are activated by brain-derived neurotrophic factor (BDNF) via *trk* B receptors and are required for a very slow depolarization in hippocampal pyramidal neurons [78]. Since TRPC3 is a downstream effector of BDNF-*trk*B signaling pathway that is thought to play a role in epilepsy, one would expect that TRPC3 should play a significant role in pilocarpine-induced seizures and neuronal cell death. The highest expression level of TRPC6 is detected in dentate granule (DG) cells. Since the mossy fiber (the afferent of DG cells) often undergoes sprouting during epileptogenesis, one might expect that TRPC6 would play an important role in the generation of seizures. We are currently investigating the role of TRPC3, 6, and 7 in the hippocampal circuitry and hopefully these studies will yield mechanistic insights regarding the generation and propagation of pilocarpine-induced acute seizures. Furthermore, TRPC3, 6, and 7 may also be involved in modulating excitotoxicity. Recent studies suggest that TRPC3 and TRPC6 may have opposite roles: Activation of TRPC3 channels contributes to excitotoxicity whereas activation of TRPC6 reduces excitotoxicity [79,80,81]. However, further studies are needed to confirm these findings since the specificity of drugs targeting TRPC3 and TRPC6 used in these studies remains questionable.

## 8. Conclusions

TRPC channels have emerged as important players in seizure and excitotoxicity: Heteromeric TRPC1/4 channels clearly play a role in excitotoxicity in the lateral septum and the CA1 area of the hippocampus; homomeric TRPC5 channels appear to play a critical role in the generation of seizures as well as excitotoxicity. Members of the TRPC3/6/7 subgroup also may contribute to both, although the underlying mechanisms have yet to be fully delineated. TRPC channels are now viewed as highly promising molecular targets for developing novel therapeutic drugs for stroke, epilepsy and head trauma. Although recent progress in drug developments has been made, a truly selective drug against any TRPC channels has yet to emerge. The wide expression of some TRPC members such as TRPC1 also complicates the matter as TRPC1 expression in different cell populations may have distinct and contradictory roles. Thus, further comprehensive studies of the functional roles of TRPC family members are critical for future drug development.
